# Coupling Between Electrons and Charge Density Wave Fluctuation and its Possible Role in Superconductivity

**DOI:** 10.1002/advs.202406043

**Published:** 2024-09-05

**Authors:** Yeonghoon Lee, Yeahan Sur, Sunghun Kim, Jaehun Cha, Jounghoon Hyun, Chan‐young Lim, Makoto Hashimoto, Donghui Lu, Younsik Kim, Soonsang Huh, Changyoung Kim, Shinichiro Ideta, Kiyohisa Tanaka, Kee Hoon Kim, Yeongkwan Kim

**Affiliations:** ^1^ Department of Physics Korea Advanced Institute of Science and Technology Daejeon 34141 Republic of Korea; ^2^ Quantum Technology Institute Korea Research Institute of Standards and Science Daejeon 34113 Republic of Korea; ^3^ Department of Physics and Astronomy Center for Novel States of Complex Materials Research Seoul National University Seoul 08826 Republic of Korea; ^4^ Department of Physics Ajou University Suwon 16499 Republic of Korea; ^5^ Stanford Synchrotron Radiation Light Source Stanford Linear Accelerator Center Menlo Park CA 94025 USA; ^6^ Center for Correlated Electron Systems Institute for Basic Science Seoul 08826 Republic of Korea; ^7^ Department of Physics and Astronomy Seoul National University Seoul 08826 Republic of Korea; ^8^ Ultra Violet Synchrotron Orbital Radiation Institute for Molecular Science Myodaiji Okazaki 444–8585 Japan; ^9^ Hiroshima Synchrotron Radiation Center Hiroshima University Higashi‐Hiroshima 739‐0046 Japan; ^10^ Institute of Applied Physics Seoul National University Seoul 08826 Republic of Korea

**Keywords:** angle‐resolved photoemission spectroscopy, charge density wave, superconductivity

## Abstract

In most charge density wave (CDW) systems of different material classes, ranging from traditional correlated systems in low‐dimension to recent topological systems with Kagome lattice, superconductivity emerges when the system is driven toward the quantum critical point (QCP) of CDW via external parameters of doping and pressure. Despite this rather universal trend, the essential hinge between CDW and superconductivity has not been established yet. Here, the evidence of coupling between electron and CDW fluctuation is reported, based on a temperature‐ and intercalation‐dependent kink in the angle‐resolved photoemission spectra of 2*H*‐Pd_x_TaSe_2_. Kinks are observed only when the system is in the CDW phase, regardless of whether a long‐ or short‐range order is established. Notably, the coupling strength is enhanced upon long‐range CDW suppression, albeit the coupling energy scale is reduced. Interestingly, the estimation of the superconducting critical temperature by incorporating the observed coupling characteristics into McMillan's equation yields results closely resembling the known values of the superconducting dome. The results thus highlight a compelling possibility that this new coupling mediates Cooper pairs, which provides new insights into the competing relationship not only for CDW but also for other competing orders.

## Graphical abstract

This study presents pioneering evidence that, beyond phonons, the charge density wave excitation mode couples with electrons, potentially serving as a mediator for Cooper pairs. This discovery not only provides a new perspective on superconductivity but also introduces a novel framework for understanding competing behaviors of superconductivity with various orderings, by exploring the energy scale and the coupling strength.

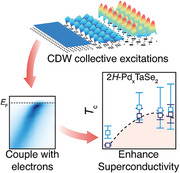

## 1 Introduction

It is widely documented that superconductivity emerges near the quantum critical point (QCP) of symmetry‐broken phases.^[^ ^1^, ^2^, ^3^, ^4^, ^5^, ^6^, ^7^, ^8^, ^9^ ^]^ This competing behavior, or the presence of a competing order, has led to notable speculation that quantum fluctuations of the order could play a role in superconductivity formation, thereby pairing electrons into Cooper pairs. For instance, spin fluctuation has been considered a pairing mediator in cuprate, iron‐based, and heavy fermion superconductors where spin ordering competes with superconductivity.^[^ ^10^, ^11^, ^12^, ^13^, ^14^, ^15^, ^16^ ^]^ A CDW, an ordering of itinerant charge carriers, also exhibits competing behavior with the superconductivity.^[^ ^1^, ^2^, ^3^, ^4^, ^5^, ^6^, ^7^, ^8^, ^9^ ^]^ Therefore, similar to the other cases, it is natural to speculate that a CDW‐associated low‐energy excitation could pair the electrons and induce the superconductivity, which has not been sufficiently visited.

**Figure 1:**
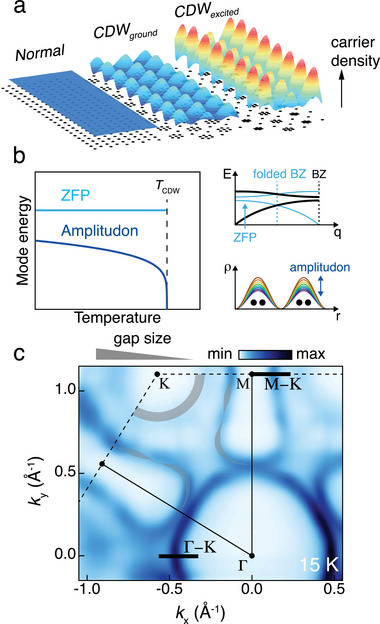
Schematics of CDW‐related excitations and the Fermi surface of 2*H*‐TaSe_2_. a) Schematics of normal, CDW ground, and CDW excited states. b) Schematics of the CDW‐related mode energy versus the temperature. The CDW amplitudon tends to soften with increasing temperature, while the ZFP energy remains constant. c) Fermi surface of 2*H*‐TaSe_2_ at 15 K, and CDW‐gap‐opened region in the Γ–K and M–K lines (the gray area; the width indicates the relative CDW gap size). The regions of interest, the Γ–K and M–K high‐symmetry lines, are indicated with black bars.

The first step in unveiling the competing behavior between CDW and superconductivity is to investigate whether electrons indeed couple with CDW‐originated excitations (**Figure** **1**a). Candidates include the zone‐folded phonon (ZFP) due to a reduced translational symmetry and collective CDW excitation modes of amplitude oscillation (amplitudon) and phase alternation (phason). Once electron couples with CDW‐originated excitations, specifically with the amplitudon (e‐amp coupling), this should be manifested in the low‐energy electronic structure. Normally, when an electron couples with a bosonic mode such as the phonon (e‐ph coupling), abrupt renormalization of electron band dispersion so‐called kink occurs, which determines the energy scale of the coupled mode and coupling strength.^[^ ^17^, ^18^, ^19^ ^]^ In contrast to e‐ph coupling, the kink attributable to CDW‐originated excitations should be temperature‐dependent, especially the energy scale, since the energy scale of amplitudon is proportional to the CDW order parameter, which reduces to zero at the CDW transition temperature *T*_CDW_ (Figure 1b).

In this work, we can capture such temperature‐dependent kinks, the smoking‐gun evidence of e‐amp coupling, in the low energy electronic structure of the representative CDW system, 2*H*‐TaSe_2_. Further, the systematic investigation across the phase diagram spanned by Pd intercalation reveals that, when the system approaches the QCP of CDW, the e‐amp coupling is strengthened, which successfully explains the superconductivity enhancement near the QCP. Our work thus implies a plausible role of this coupling in the superconductivity, bridging CDW and superconductivity.

## 2 Results

### 2.1 Proper Momentum Positions for Investigating Kink

To explicitly search for these temperature‐dependent kinks, alternative temperature‐dependent band renormalizations associated with the CDW transition should be avoided. Upon cooling from the normal phase, TaSe_2_ first enters the incommensurate CDW (ICCDW) phase at *T*_ICC_, and the transition into commensurate CDW (CCDW) occurs at a lower temperature, i.e., *T*_CC_. Between these two transitions, there is a region where CCDW and ICCDW coexist, referred to as the coexisting phase.^[^ ^20^, ^21^ ^]^ This series of transitions leaves a footprint in the electronic structure, such as the pseudogap, band folding, and CDW gap.^[^ ^22^, ^23^, ^24^ ^]^ To avoid those renormalizations, especially the complicated gap opening at the Fermi level, possible kinks in the band along the Γ–K and M–K lines were investigated (Figure 1c).

### 2.2 Electron‐Amplitudon Kink via Temperature‐Dependent Measurements

**Figure 2:**
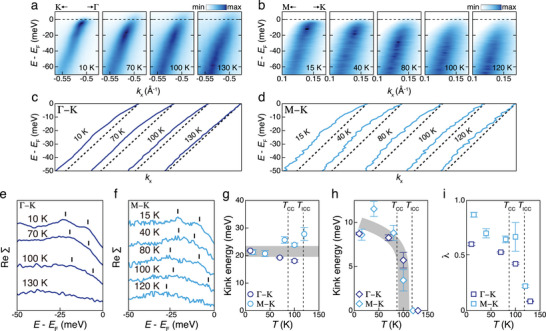
Temperature‐dependent kinks in 2*H*‐TaSe_2_. a,b) Temperature‐dependent Γ–K (a) and M–K (b) high‐symmetry cuts. c,d) The peak positions obtained via MDC fitting of the Γ–K (c) and M–K (d) cuts. e,f) Temperature‐dependent real part of the self‐energy with offsets obtained by subtracting the estimated bare band dispersions (the dashed lines in (c) and (d)) from the peak positions (the colored solid lines in (c) and (d)) along the Γ–K (e) and M–K (f) lines. g,h) Higher (g) and lower (h) kink energy levels obtained from fitting to a continuous function consists of several linear lines (the black bars in (e) and (f)). The gray line in (g) is the average kink energy level at the lowest temperature while that in (h) is the data fitted to the softening function. i) Coupling constant obtained from the slope of Re**Σ** at *E*_F_.

**Figure** **2** shows band dispersion along the Γ–K and M–K lines at different temperatures. At low temperatures (Figure 2a,b), kinks are relatively clearly observed in both the Γ–K and M–K lines, respectively. The peak positions obtained via momentum distribution curve (MDC) fitting (see Supporting Information) reveal the presence of kinks at two different binding energies in both high‐symmetry lines (Figure 2c,d). The low‐energy dispersion deviates from the estimated bare band dispersion at the two binding energies (the dashed lines, see Supporting Information for estimation). Notably, in both lines, low‐energy kinks disappear at a high temperature of 130 K, which is above *T*_CC_ and *T*_ICC_.

To characterize these kinks, the real part of self‐energy (ReΣ) is extracted as shown in Figure 2e,f for the Γ–K and M–K lines, respectively, by subtracting the bare band dispersion from fitted peak positions.^[^ ^17^ ^]^ The energy points of sudden slope change determine the kink energy scales, 9 meV for low‐energy kink and 22 meV for high‐energy kink, as identified by the black vertical lines (see Supporting information for validity of extracted ReΣ and determination of kink energy scales). The energy scales of the observed kinks in both the Γ–K and M–K lines are almost identical. With high‐energy kinks, the kink energy scale is much larger than the known collective excitations and does not freeze to zero (Figure 2g), suggesting that high‐energy kinks originate from either the ordinary phonon or ZFP. In sharp contrast, with increasing temperature, the lower‐energy kinks exhibit softening to an energy scale ranging from 4–6 meV before finally disappearing (Figure 2h). The thermal effect–the broadening of electron spectral weight itself and the weakening of coupling strength, could cause the disappearance of the kink with low energy scale at high temperatures. However, the thermal effect does not provoke the change in the kink energy (Figure S8, Supporting Information). Thus, the observed softening requires another origin, which is not expected for the phonon but is adequate for the CDW amplitudon.^[^ ^25^ ^]^ Indeed, the observed energy scale and associated softening behavior closely match those of the A_1g_ amplitudon measured through Raman spectroscopy^[^ ^26^, ^27^, ^28^ ^]^, suggesting the e‐amp coupling origin for the lower‐energy kinks. Further, no other candidates fit into the observations; i) there is no optical phonon with energy less than 15 meV^[^ ^26^, ^27^, ^28^, ^29^, ^30^ ^]^, ii) the acoustic phonons cannot explain the isotropic nature of low‐energy kink (Figure S10, Supporting Information and related texts), iii) sudden drop of the coupling strength above *T*_CDW_ does not fit phonons (Figure 2i), iv) the CDW is the only order which generates the collective excitation mode other than phonons, v) the CDW phason energy does not severely change while increasing temperature.^[^ ^26^, ^27^, ^28^ ^]^ Therefore, we conclude these low‐energy (high‐energy) kinks as e‐amp (e‐ph) kinks.

**Figure 3:**
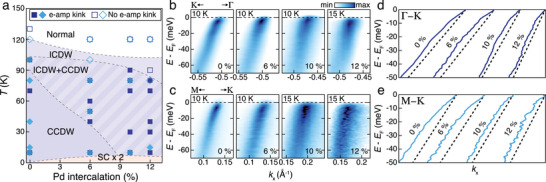
Phase diagram and kinks of 2*H*‐Pd_x_TaSe_2_. a) Estimated phase diagram of 2*H*‐Pd_x_TaSe_2_. The dark blue filled (empty) squares indicate where e‐amp kinks are (not) detected in the Γ–K line, while the light blue diamond symbols are for the M–K line. b,c) Intercalation‐dependent Γ–K (b) and M–K (c) high symmetry cuts. d,e) The peak positions of the Γ–K (d) and M–K (e) line obtained through MDC fitting.

Fitting the temperature dependence of e‐amp kinks with the formula ω (*T*) =  ω(0)(1 − *T*/*T*_CDW_)^0.19^ yields a *T*_CDW_ value of 102 K (the gray curve in Figure 2h). The exponent value of 0.19 was retrieved from the previous Raman study.^[^ ^26^ ^]^ It should be noted that, instead of *T*_CC_, the fitted *T*_CDW_ value reaches 107 K, where the coexisting phase ends, suggesting that e‐amp kinks persist up to the coexisting phase, where CCDW is expected to establish a short‐range order. This somewhat unexpected behavior was clarified further through systematic investigation of the kinks across the phase diagram derived via Pd intercalation, as shown in **Figure** **3**. The filled markers in Figure 3a indicate the points where e‐amp kinks are observed. The empty markers indicate the absence of kinks. This suggests that e‐amp kinks persist in the Pd‐intercalated system up to the coexisting phase at all intercalation levels, even at high levels where the long‐range CCDW no longer remains stable at any temperature. Moreover, the suppression of the e‐amp kink energy is repeated with increasing temperature at each level (see Supporting Information). This strongly suggests that the amplitudon persists up to the coexisting phase instead of *T*_CC_, which will be discussed later.

### 2.3 Evolution of Kink Energy and Coupling Strength Upon Pd Intercalation

**Figure 4:**
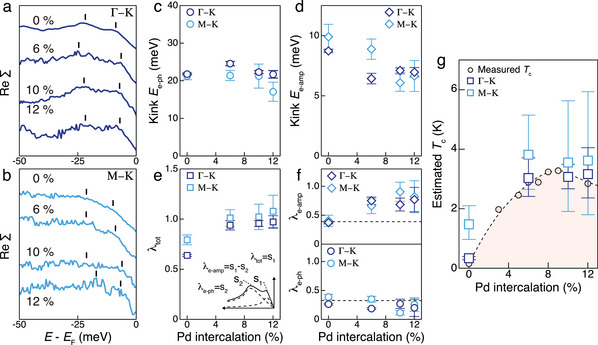
Intercalation‐dependent kink analysis. a,b) The real part of the self‐energy with offsets for Γ–K (a) and M–K (b) lines. c,d) Intercalation dependence of the e‐ph (c) and e‐amp (d) kink energies which are indicated as black bars in (a) and (b). e,f) Total, e‐amp, and e‐ph coupling constant as a function of intercalation level. The inset in (e) shows how each coupling constant is extracted from the real part of the self‐energy. g) Estimated *T*_c_ from the McMillan's equation with obtained parameters. The filled circles are the *T*_c_ obtained by the transport measurement in ref. [2], and the dashed line is a guide for the eye.

To trace the evolution of kinks upon Pd intercalation, the lowest‐temperature dispersions are compared between the different intercalation levels, as shown in Figure 3b,c. The spectral weight tends to broaden, presumably due to the induced inhomogeneity. Nevertheless, the precise band dispersions obtained through MDC fitting (Figure 3d,e) clearly illustrate both e‐amp and e‐ph kinks, respectively, persisting up to the highest level in Pd_0.12_TaSe_2_. Since Pd intercalation is expected to modify the bare band dispersion, further analysis was carried out with ReΣ, as shown in **Figure** **4**a,b for both the Γ–K and M–K lines, respectively. The extracted ReΣ indicates that the e‐ph kink energy remains almost intact against Pd intercalation (Figure 4c). This seems reasonable since Pd intercalation between TaSe_2_ layers should hardly affect the phonon.^[^ ^2^ ^]^ Meanwhile, e‐amp kinks exhibit progressive softening upon Pd intercalation (Figure 4d). This is the expected trend since the magnitude of the CDW order parameter decreases upon Pd intercalation, as evidenced by the reduced *T*_CC_, which eventually disappears at a high intercalation level. Consistently, amplitudon softening upon CDW phase suppression was observed in a similar 2*H*‐TaS_2_ system, wherein the suppression is derived by pressure.^[^ ^31^ ^]^

Next, we examined the strength of each coupling upon intercalation. First, the total coupling strength λ_tot_ was estimated from the slope of ReΣ at *E*_F_, by definition. A monotonic increase of λ_tot_ was revealed with increasing intercalation level (Figure 4e). This tendency is consistent with that extracted from the transport property, which accounts for the enhancement of superconducting transition temperature *T*_c_.^[^ ^2^, ^3^ ^]^ As two different couplings contribute to λ_tot_, the strength of each coupling was separated by considering the slope of ReΣ at higher binding energy levels between the e‐amp and e‐ph kinks, as shown in the inset of Figure 4e and Figure S11 (Supporting Information). The resulting separation in Figure 4f reveals a different trend between λ_e‐amp_ and λ_e‐ph_. Interestingly, only λ_e‐amp_ increases while λ_e‐ph_ remains almost constant, indicating that λ_tot_ enhancement is dominated by λ_e‐amp_. In the beginning, λ_e‐amp_ is comparable to λ_e‐ph_, at a value ≈0.4. Over time, λ_e‐amp_ approaches a value ranging from 0.9–1.0, more than twice that of λ_e‐ph_, at the highest level.

The increase in λ_tot_, primarily attributed to λ_e‐amp_, leads to the fascinating conjecture that e‐amp coupling plays a specific role in *T*_c_ enhancement. Figure 4g shows *T*_c_ estimated by incorporating the collected coupling information into the McMillan's equation^[^ ^32^ ^]^ $T_{c}=\frac{\Theta_{D}}{1.45}\;\exp\left(-\frac{1.04(1+\lambda )}{\lambda -\mu^{*}\left(1+0.62\lambda \right)}\right)$. The average value of the two coupling energy scales at each intercalation level was substituted as the Debye temperature, along with λ_tot_ at each level. The Coulomb pseudopotential µ^*^ was assumed as 0.25 and remained fixed for every level as the electron density is expected to maintain upon Pd intercalation. The estimated *T*_c_ value agreed well with the *T*_c_ value retrieved from transport measurements.^[^ ^2^, ^3^ ^]^ This consistency ensures that the couplings included in λ_tot_ constitute the source of superconductivity—the pairing mediator. Furthermore, the dominance of λ_e‐amp_ suggests that e‐amp coupling is essential between these two couplings. We note that under e‐ph coupling only, an extremely low *T*_c_ value is expected so that *T*_c_ enhancement upon Pd intercalation cannot be explained.

## 3 Discussion

The preceding results—the discovery of the signature of e‐amp coupling and corresponding evolution toward the QCP—highlight the unprecedented role of CDW fluctuation in superconductivity formation, pairing electrons by amplitudons. Yet, the e‐amp coupling has been traced only at the limited momentum positions, due to the previously mentioned complex gap opening. Still, the results could be representative of the possible coupling at other positions and for the formation of superconductivity, since expected optical‐phonon‐like flat amplitudon dispersion suggests a weak momentum dependence of e‐amp coupling. Further, the known *s*‐wave symmetry of superconductivity entails a rather isotropic interaction source for the superconductivity.^[^ ^33^, ^34^, ^35^ ^]^

Not only the possible role of e‐amp coupling on the superconductivity, but our results also suggest a way to parametrically describe competing behavior. Our results reveal two trends in e‐amp coupling as the system approaches the QCP of CDW. One trend entails amplitudon energy softening, and the other trend involves e‐amp coupling strengthening. According to McMillan's equation, a reduction in amplitudon energy linearly reduces *T*_c_ since the coupling energy determines the Debye temperature. Stronger e‐amp coupling exponentially enhances *T*_c_, as the coupling strength functions as an exponent. Therefore, in a particular well‐balanced region, an increase in coupling strength can overcome amplitudon energy reduction and produce a higher *T*_c_ value, which will eventually decrease to zero as the amplitudon energy becomes infinitesimal. Therefore, the dome‐shaped superconducting phase near the QCP of the CDW can be regarded as a balance between these two factors.

The nature of these two trends must be elucidated. In the case of amplitudon energy reduction, e‐amp kinks persist up to the coexisting phase where CCDW supposedly establishes a short‐range order only. This indicates that the amplitudon can be excited from a short‐range order or even with a certain degree of instability, similar to the case of the magnon observed without long‐range spin ordering.^[^ ^36^, ^37^ ^]^ However, in this case, a lower energy scale is expected than that of the excitation out of the well‐stabilized long‐range order, as mentioned above. Next, regarding the coupling strength, the Eliashberg–McMillan theory gives the dimensionless coupling constant as $\lambda \;=\int \frac{d\omega \alpha^{2}\left(\omega \right)F\left(\omega \right)}{\omega }\;=\frac{N\left(0\right)\left\langle g^{2}\right\rangle }{M\langle \omega^{2}\rangle }$ where *N*(0) is the electronic density of states (DOS) at *E*_F_, *g* is the electronic matrix element, and ω is the phonon frequency.^[^ ^32^ ^]^ According to the above definition, it can be easily expected that the suppression of CDW order (and so does CDW gap) recovers DOS at the Fermi surface *N*(0), leading to the coupling strength enhancement. Also, the softening of the amplitudon energy, which reduces 〈ω^2^〉, can strengthen the coupling. Therefore, the enhancement of coupling strength would be a natural consequence of CDW suppression. Still, a further theoretical and experimental investigation is needed to identify the actual mechanism of the enhancement, including other possible contributions such as the spectral weight of the amplitudon and van Hove singularity.^[^ ^3^ ^]^

This compelling scenario of superconductivity by e‐amp coupling requires further inquiries for substantiation. Future research should also address related questions, such as how an electron can interact with self‐governed order fluctuations, handling multiple couplings of different origins in electron pairing, the case of other competing orders in different systems, etc. We believe that these efforts will establish the microscopic mechanism behind the competing behavior, thereby elucidating the nature of the superconducting mechanism. The present results lay the foundation for such effort and, further, would be also informative in establishing the position of the CDW in cuprate superconductors and the connection between the non‐trivial nature of both CDW and superconductivity in recent Kagome systems.

## 4 Experimental Section

Single crystals of the pristine and Pd‐intercalated 2*H*‐Pd_x_TaSe_2_ samples were grown by the chemical vapor transport (CVT) method. The transport agent of the pristine samples was I_2_, while that of the intercalated samples was SeCl_4_. In the case of the intercalated 2*H*‐Pd_x_TaSe_2_, the solid‐state reaction method was used before the CVT to synthesize the polycrystalline samples with different *x* values. The details of the 2*H*‐Pd_x_TaSe_2_ sample growth and the characterization are described in ref. [2].

High‐resolution ARPES was conducted with synchrotron‐radiation light sources of beamline 5‐2 at the Stanford Synchrotron Radiation Lightsource (SSRL) of the Stanford Linear Accelerator Center and of beamline 5U at the Ultra Violet Synchrotron Orbital Radiation (UVSOR) of the Institute of Molecular Science. Supplementary high‐resolution ARPES spectrum was obtained at the Korea Research Institute of Standards and Science (KRISS), with a He lamp as a light source. Time‐of‐flight ARPES was conducted with a laser light source at the Center for Correlated Electron Systems (CCES) of the Institute of Basic Science (IBS). A SCIENTA DA30 electron analyzer was used at SSRL to detect photoelectrons. The utilized incident photon energies for the main data were 48 and 30 eV. The samples were cleaved in situ at a temperature lower than 15 K under ultrahigh vacuum better than 1 × 10^−10^ Torr. The energy resolution was 4 meV for 30 eV and 10 meV for 48 eV, estimated with the reference gold spectrum.

## Conflict of Interest

The authors declare no conflict of interest.

## Author Contributions

Y.K.K. conceived the work. Y.L., S.K., J.C., J.H., C.L., and Y.K.K. performed the ARPES measurements with support from M.H. and D.L. at SSRL, from S.I. and K.T. at UVSOR, and from Y.S.K., S.H., C.K. at IBS CCES. J.C. and S.H. grew the 2H‐TaSe2 single crystals, and Y.S. and K.H.K. grew the 2H‐PdxTaSe2 single crystals. Y.L. and Y.K.K. analyzed the ARPES data and all authors discussed the results. Y.L. and Y.K.K. wrote the manuscript in consultation with all authors.

## Supporting information

Supporting Information

## Data Availability

The data that support the findings of this study are available from the corresponding author upon reasonable request.
