# Supplementary material for: Coupling Between Electrons and Charge Density Wave Fluctuation and its Possible Role in Superconductivity
Source: Adv Sci (Weinh). 2024 Sep 5;11(41):2406043. doi: 10.1002/advs.202406043 (PMC11538642; doi:10.1002/advs.202406043)
Supplement: Supplementary file 1 — Supporting Information [file ADVS-11-2406043-s001.docx]

**Coupling between electrons and charge density wave fluctuation and its possible role in superconductivity**

Yeonghoon Lee^1,2^, Yeahan Sur^3^, Sunghun Kim^1,4^, Jaehun Cha^1^, Jounghoon Hyun^1^, Chan-young Lim^1^, Makoto Hashimoto^5^, Donghui Lu^5^, Younsik Kim^6,7^, Soonsang Huh^6,7^, Changyoung Kim^6,7^, Shinichiro Ideta^8,9^, Kiyohisa Tanaka^8^, Kee Hoon Kim^3,10^, Yeongkwan Kim^1^*

*^1^Department of Physics, Korea Advanced Institute of Science and Technology, Daejeon 34141, Republic of Korea.*

*^2^Quantum Technology Institute, Korea Research Institute of Standards and Science, Daejeon 34113, Republic of Korea.*

*^3^Center for Novel States of Complex Materials Research, Department of Physics and Astronomy, Seoul National University, Seoul 08826, Republic of Korea.*

*^4^Department of Physics, Ajou University, Suwon 16499, Republic of Korea.*

*^5^Stanford Synchrotron Radiation Light Source, Stanford Linear Accelerator Center, Menlo Park, CA, 94025, USA.*

*^6^Center for Correlated Electron Systems, Institute for Basic Science, Seoul 08826, Republic of Korea.*

*^7^Department of Physics and Astronomy, Seoul National University, Seoul 08826, Republic of Korea.*

*^8^Ultra Violet Synchrotron Orbital Radiation, Institute for Molecular Science, Myodaiji, Okazaki 444-8585, Japan.*

*^9^Hiroshima Synchrotron Radiation Center, Hiroshima University, Higashi-Hiroshima 739-0046, Japan.*

*^10^Institute of Applied Physics, Seoul National University, Seoul 08826, Republic of Korea.*

**e-mail:* [*yeongkwan@kaist.ac.kr*](mailto:yeongkwan@kaist.ac.kr)

Momentum distribution curve fitting method and results

To determine the precise peak positions and widths from the ARPES spectra, we have fitted the momentum distribution curves (MDCs) for all data sets. For the case of M-K and Γ-M high symmetry lines of 2*H*-TaSe_2_, there are multiple bands (four for M-K, and two for Γ-M) which can affect the fitting, so all corresponding peaks were included in the fit function. In Figure S1 – S4, MDC fitting results of the all datasets used in the main text are presented. It is clear that all the fit lines (black solid lines) closely reproduce the raw data (colored open circles).

**Estimation of bare band dispersion**

To estimate the bare band dispersion, the peak positions obtained by MDC fitting are fitted with a polynomial function. Then, the bare band dispersion is determined as a linear line connecting two peak positions, one at *k*_F_ and the other at the high binding energy of the highest temperature. In Figure S5, the extended peak positions obtained by MDC fitting and the estimated bare band is plotted.

**Fermi surface of Pd-intercalated 2*H*-TaSe_2_**

In Figure S6, the Fermi surfaces of each 2*H*-Pd_x_TaSe_2_ (x=0.06, 0.10 and 0.12) are displayed. As expected, the band folding feature and the CDW gap opening were disappeared at high intercalation level. It demonstrates that the long-range CDW order is completely suppressed at intercalation level higher than 10 %.

**Temperature- and intercalation-dependent kink**

The temperature and intercalation dependence of kinks are presented with the real part of self-energies (ReΣ), given in Figure S7. The same analysis procedure used for Figure 2 in the main text was applied for all cases. For both Γ-K and M-K cases, the line shape analysis of peak position evidently indicates the existence of temperature-dependent kink. ReΣ can be extracted as mentioned in the main text, and from those, kink energies were estimated. Particularly, in the case of low intercalation level, the softening behavior for the e-amp kink upon increasing temperature is well reproduced.

**Simulation on the thermal broadening effect**

To check whether thermal broadening effect interferes the detection of the kink with small energy scale of around 10 meV at high temperature, the simulation for the temperature-dependent kink was performed. The bare band was assumed to be linear and fixed. The energy resolution effect is introduced by convoluting the generated spectra with Gaussian function of width 10 meV, which is the largest value for the main data. The energy scale of the kink is set to 10 meV for all temperature to solely examine the thermal broadening effect. As given in Figure S8, a kink is apparent even in the high-temperature case. Further, a kink can be more clearly revealed by the fitting the simulated spectra with MDC. Based on the simulation, it can be concluded that the kink structure cannot be erased by the thermal broadening effect.

**Temperature dependence of the electron-phonon coupling**

To estimate the temperature dependence of band renormalization produced by electron-phonon coupling, we calculated the renormalization of band including the temperature dependence of the self-energy. Normally, there are three main contributions to the self-energy: ${\Sigma"}_{e-ph}$ (electron-phonon scattering), ${\Sigma"}_{e-e}$(electron-electron scattering), and ${\Sigma"}_{e-df}$ (electron-defect interactions) [1-3]. It is known that ${\Sigma"}_{e-df}$ is not usually strong and serves as a constant offset, and the effect of ${\Sigma"}_{e-e}$ is small near the Fermi level and thus its temperature dependence. Therefore, only ${\Sigma"}_{e-ph}$ with offset was accounted for the temperature dependence, which has the form of

$\left| {\Sigma"}_{e-ph} \right|=\pi\hbar\int_{0}^{\omega_{max}} \alpha^{2}F\left( \omega' \right)\left[ 1-f\left( \omega-\omega^{'} \right)+2n\left( \omega^{'} \right)+f\left( \omega+\omega' \right) \right]d\omega'$ (S1)

where $\alpha^{2}F\left( \omega\right)$ is the Eliashberg coupling function, *f* is the Fermi-Dirac distribution function, and *n* is the Bose-Einstein distribution function. $\alpha^{2}F\left( \omega\right)$ is assumed to have the shape of a Debye spectrum,

$\alpha^{2}F\left( \omega\right)=\left\{ \begin{aligned} {3A\left| \omega\right|^{2}}/{\omega_{D}^{3}}, \mathrm{if} \left| \omega\right|<\omega_{D} \\ 0, \mathrm{if} \left| \omega\right|\geq\omega_{D} \end{aligned} \right.$ (S2)

and the real part of the self-energy $\Sigma'$ is retrieved using Kramers-Kronig relations.

For the kink energy, the average kink energy at the M-K line is used for the simulation, which is 24.5 meV. The constant *A* of the equation (S2) is roughly determined to $5\times{10}^{12}$ which reproduces well the actual M-K data. Note that the exact value for the constant *A* is not necessary as the overall trend of the coupling constant is not affected by it. We used the estimated bare band of M-K in Figure S5e as a bare band. Inserting all, the renormalization of band dispersion at various temperature is simulated (Figure S9). As the temperature is raised, the kink structure gradually broadens but does not disappear. The solid curve of coupling strength in Fig. S9b shows that the coupling strength reduces only by a half even at 150 K thus the kink should be visible.

**Ruling out optical and acoustic phonons as an origin of the low-energy kink**

It is easy to show that optical phonon is not the one that induces the low-energy kink as there is no optical phonon with energy less than 15 meV [4-8]. On the other hand, the case of acoustic phonon requires delicacy. The energy level of the acoustic phonon matches with the low-energy kink, and recent inelastic x-ray scattering study on 2*H*-TaSe_2_ have shown that acoustic phonon also softens toward *T*_CDW_ when the wave vector ***q*** is close to ***q***_CDW_, although it recovers its energy above *T*_CDW_ [9]. This makes it hard to exclude the possibility that e-acoustic-phonon coupling induces the low-energy kink. To rule out such possibility, additional high-resolution ARPES mapping was conducted (Fig. S10). To compensate the low statistics, which is a tradeoff for high resolution, denoising technique [10] is used to get clean spectrum (Fig. S10b). The band dispersions from MDC fitting and the resulting kink energies in Fig. S10c and S10d shows that the kink energies are almost constant upon momentum variation. If the low-energy kink is indeed from the acoustic phonon, the kink energy should soften to zero when the nesting condition is met [9, 11]. As no such anomaly is seen at any momentum, we conclude that the acoustic phonon is not the source of the low-energy kink.

**Separation method of two adjacent kinks**

To separate two different kinks avoiding intentional choice, a continuous function consisting of several linear lines is used as a fitting function (Figure S11). The vertices $a_{0}$ and $a_{2}$ are defined as $E_{\mathrm{amp}}$ and $E_{\mathrm{ph}}$, and the slopes $a_{4}$ and $a_{6}$ are defined as $\lambda_{\mathrm{tot}}$ and $\lambda_{e-ph}$. The e-amplitudon coupling constant $\lambda_{e-amp}$ is calculated by subtracting $\lambda_{\mathrm{tot}}$ and $\lambda_{e-ph}$.

To verify whether the coupling constants are properly separated, the Debye model is used to fit the real part of the self-energy $\Sigma'$. From equation (S1) and (S2), the imaginary part of the self-energy $\Sigma"$ from the e-phonon coupling can be reduced to,

$\left| {\Sigma"}_{e-ph}\left( \omega\right) \right|=\left\{ \begin{aligned} {\hbar\lambda\pi\left| \omega\right|^{3}}/\left( 3\omega_{D}^{2} \right), \mathrm{if} \left| \omega\right|<\omega_{D} \\ {\hbar\lambda\pi\omega_{D}}/3, \mathrm{if} \left| \omega\right|\geq\omega_{D} \end{aligned} \right.$, (S3)

at sufficiently low temperature [1,2]. With additional polynomial function $\beta x^{2}$ from ${\Sigma"}_{e-e}$ contribution, one can calculate the real part of the self-energy using the Kramers-Kronig transformation. Using this as a fitting function, the coupling strengths of the two kinks are separated in Figure S12. The resulting trend of the coupling strengths is consistent with our analysis; $\lambda_{\mathrm{tot}}$ and $\lambda_{e-amp}$ significantly increases upon Pd intercalation while $\lambda_{e-ph}$ does not. This result implies that the definition in Figure S11 is appropriate.

**The effect of band hybridization on coupling constant analysis**

Distinct feature of the real part of the self-energy of 2*H*-Pd_x_TaSe_2_ is that the self-energy does not converge to zero even at high binding energy regime. Indeed, the band dispersion at the lowest temperature does not meet the estimated bare band dispersion (Figure S13). The most probable explanation of this uplift is a gap induced by band hybridization. When the system is in the CDW state, the hybridization of the main band and the folded band induces a gap in the electronic structure (Figure S14). By using a simple model of gapped dispersion relation [12],

$E_{\pm}\left( k \right)=\frac{\hbar^{2}}{2m}\left( \frac{1}{4}G^{2}+\left( k-\frac{1}{2}G \right)^{2} \right)\pm\sqrt{4\left( \frac{\hbar^{2}}{2m}\left( \frac{1}{2}G \right)^{2} \right)\left( \frac{\hbar^{2}}{2m}\left( k-\frac{1}{2}G \right)^{2} \right)+U^{2}}$, (S4)

it is possible to simulate the band dispersion of 2*H*-TaSe_2_ in the CDW state (Figure S14a and b). Although this hybridization considerably renormalizes the band dispersion, its effect on coupling constant is negligible due to the parabolic feature of the renormalization (Figure S14f).

**Intercalation-dependent analysis of the imaginary part of the self-energy**

If we assume that the bare dispersion is almost linear near the Fermi level, *i.e.,* $\varepsilon\left( k \right)=vk$ , we can calculate the imaginary part of self-energy $\Sigma"$ from MDC width $FWHM=2\left| {\Sigma"\left( \omega\right)}/v \right|$. The results are plotted in Figure S15a (dark blue circles). And then, the imaginary part of the self-energy is fitted to equation (S3) with additional polynomial function $\beta x^{2}$ for ${\Sigma"}_{e-e}$ contribution [1], as the system shows Fermi liquid behavior below 30 K [13]. From the fitted parameters, it is possible to extract the total λ_tot_, e-amp λ_e-amp_, and e-ph coupling constants λ_e-ph_ (Figure S15b and c). With those parameters, we also calculated *T*_c_ separately to cross check those estimated with parameters extracted from the real part of self-energy (Figure S15d). Coupling constants were extracted with 2 different sets of imaginary parts of self-energy at each intercalation level and averaged. The tendency of the estimated *T*_c_ fits with that obtained from the real part of self-energy. although slightly larger value for the Coulomb pseudopotential μ* was used compared to the real part of the self-energy analysis. This consistency between two independent analysis supports our interpretation that the coupling constant is enhanced due to the intercalation and the major cause of the enhancement is e-amp coupling.

It is also possible to check the validity of our definition of the real part of the self-energy $\Sigma'$ using the imaginary part $\Sigma"$. If $\Sigma'$ is properly extracted, $\Sigma'$ and $\Sigma"$ should be connected by the Kramers-Kronig transformation [1,2]. In Figure S16, $\Sigma"$ obtained by Kramers-Kronig transformation is compatible with $\Sigma"$ from the MDC width. This correspondence verifies that the real part of the self-energy is well-defined.

<Reference>

[1] Hofmann, Ph., Sklyadneva, I. Yu., Rienks, E. D. L. & Chulkov, E. V., Electron-phonon coupling at surfaces and interfaces. *New J. Phys.* **11**, 125005 (2009).

[2] Grimvall, G. *The electron-phonon interaction in metals.* (North-Holland Publishing Company, Amsterdam, New York, Oxford, 1981).

[3] Damascelli, A., Hussain, Z. & Shen, Z.-X. Angle-resolved photoemission studies of the cuprate superconductors. *Rev. Mod. Phys.* **75**, 473 (2003).

[4] Hill, H. M. *et al.* Phonon origin and lattice evolution in charge density wave states. *Phys. Rev. B* **99**, 174110 (2019).

[5] Lin, D. *et al.* Patterns and driving forces of dimensionality-dependent charge density waves in 2*H*-type transition metal dichalcogenides. *Nat. Commun.* **11**, 2406 (2020).

[6] Steigmeier, E. F., Harbeke, G., Auderset, H. & DiSalvo, F. J. Softening of charge density wave excitations at the superstructure transition in 2*H*-TaSe_2_. *Solid State Commun.* **20**, 667-671 (1976).

[7] Lucovsky, G. *et al.* Reflectivity studies of Ti- and Ta-dichalcogenides: phonons. *Solid State Commun.* **19**, 303-307 (1976).

[8] Ge, Y. & Liu, A. Y. Effect of dimensionality and spin-orbit coupling on charge-density-wave transition in 2*H*-TaSe_2_. *Phys. Rev. B* **86**, 104101 (2012).

[9] Shen, X. *et al.* Precursor phase with full phonon softening above the charge-density wave phase transition in 2*H*-TaSe_2_. *arXiv*:2207.11298 (2022).

[10] Kim, Y. *et. al.* Deep learning-based statistical noise reduction for multidimentsional spectral data. *Rev. Sci. Instrum.* **92**, 073901 (2021).

[11] Grüner, G. *Density waves in solids Ch. 6* (Perseus Publishing, Cambridge, 1994).

[12] Kittel, C. Introduction to Solid State Physics Global Ed. Ch. 7 (John Wiley & Sons, New York, 2005).

[13] Bhoi, D. *et al.* Interplay of charge density wave and multiband superconductivity in 2*H*-Pd_x_TaSe_2_. *Sci. Rep.* **6**, 24068 (2016).


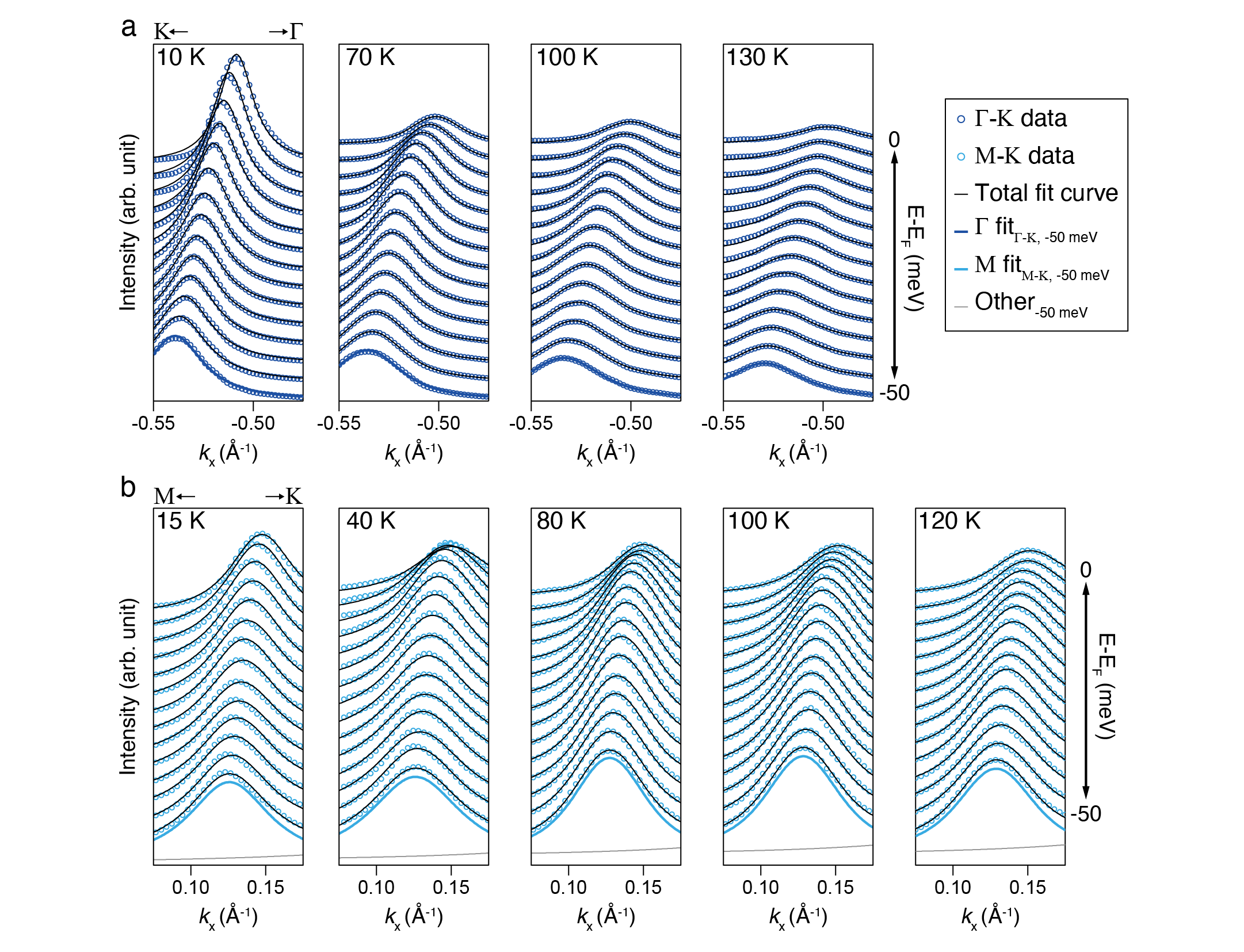


Supplementary Figure S1.

a, b) MDC fitting results of pristine TaSe_2_ at the Γ-K (a) and M-K (b) high symmetry lines with various temperatures and binding energies. Dark/light blue empty circles are the raw data, black solid lines are total fit curve including all necessary terms. Dark/light blue solid lines are single-peak fittings for each spectrum, and gray lines in (b) are additional peak of K band right near the M band where the peak position locates outside of the plotted momentum range. The energy step of MDC stack is approximately 3.6 meV; some data points in both energy and momentum directions are not plotted for the better visualization.


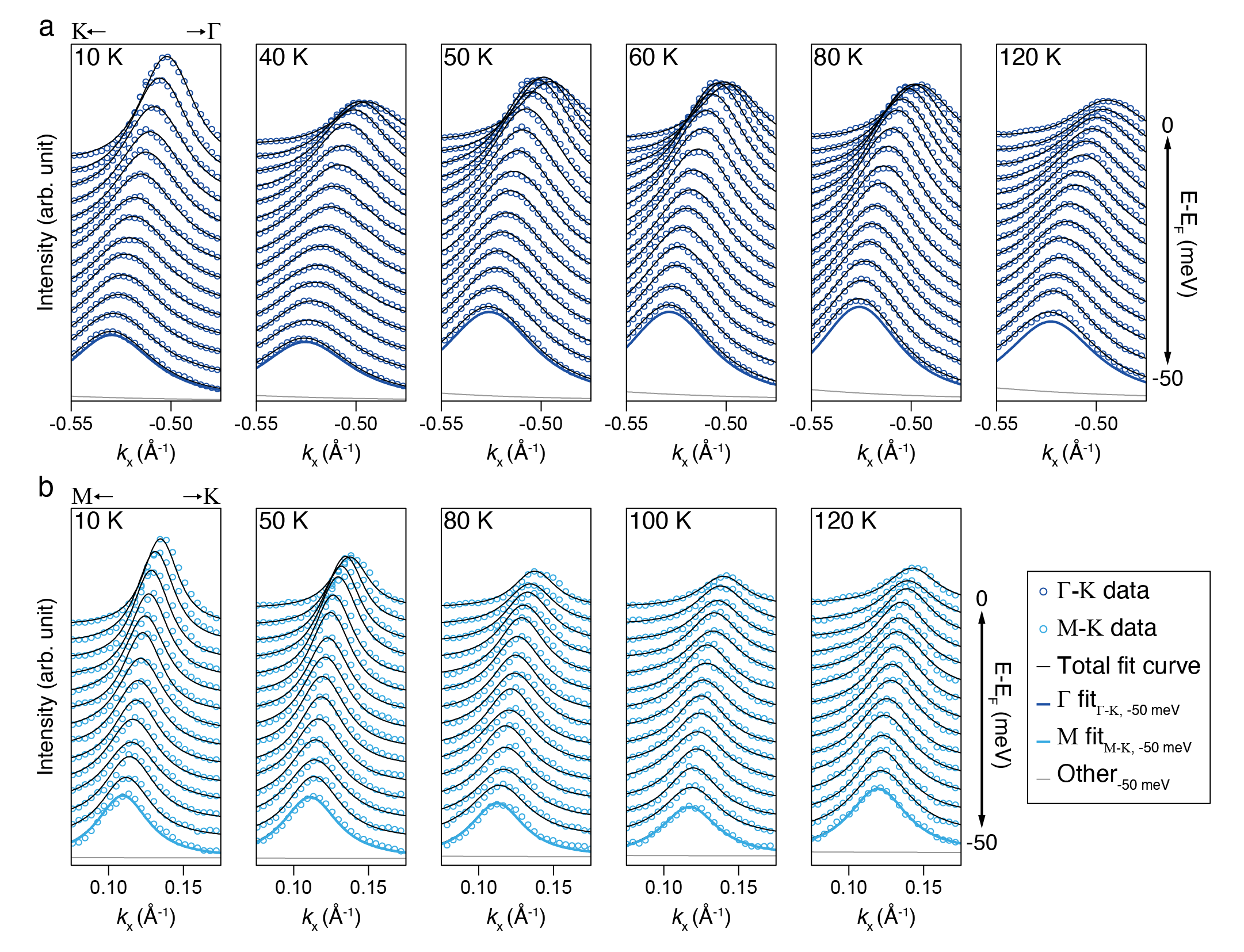


Supplementary Figure S2.

a, b) MDC fitting results of Pd_0.06_TaSe_2_ at the Γ-K (a) and M-K (b) high symmetry lines with various temperatures and binding energies. Dark/light blue empty circles are the raw data, black solid lines are total fit curve including all necessary terms. Dark/light blue solid lines are single-peak fittings for each spectrum, and gray lines are additional peaks where the peak positions locate outside of the plotted momentum range. The energy step of MDC stack is approximately 3.6 meV; some data points in both energy and momentum directions are not plotted for the better visualization.


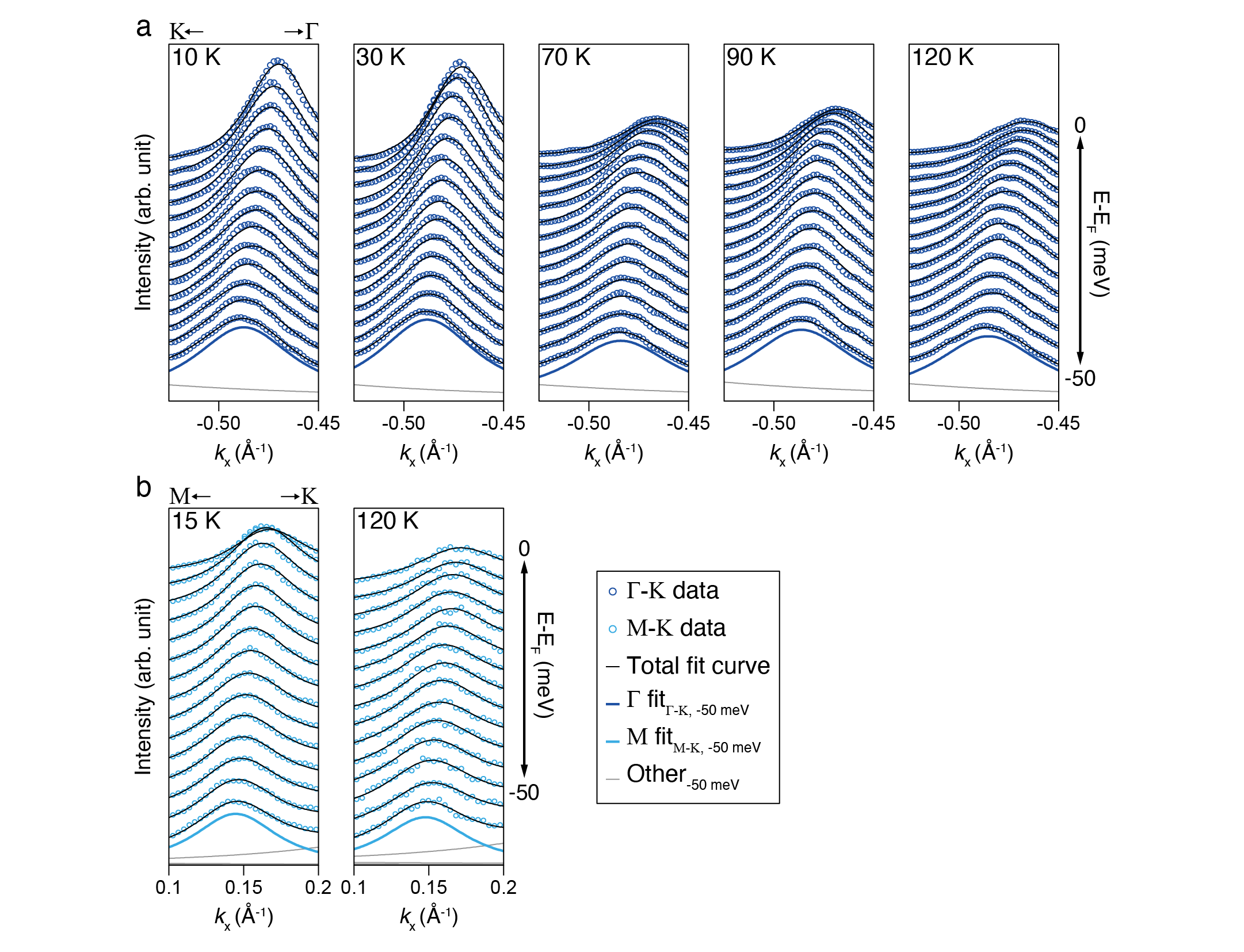


Supplementary Figure S3.

a, b) MDC fitting results of Pd_0.10_TaSe_2_ at the Γ-K (a) and M-K (b) high symmetry lines with various temperatures and binding energies. Dark/light blue empty circles are the raw data, black solid lines are total fit curve including all necessary terms. Dark/light blue solid lines are single-peak fittings for each spectrum, and gray lines are additional peaks where the peak positions locate outside of the plotted momentum range. The energy step of MDC stack is approximately 3.6 meV; some data points in both energy and momentum directions are not plotted for the better visualization.


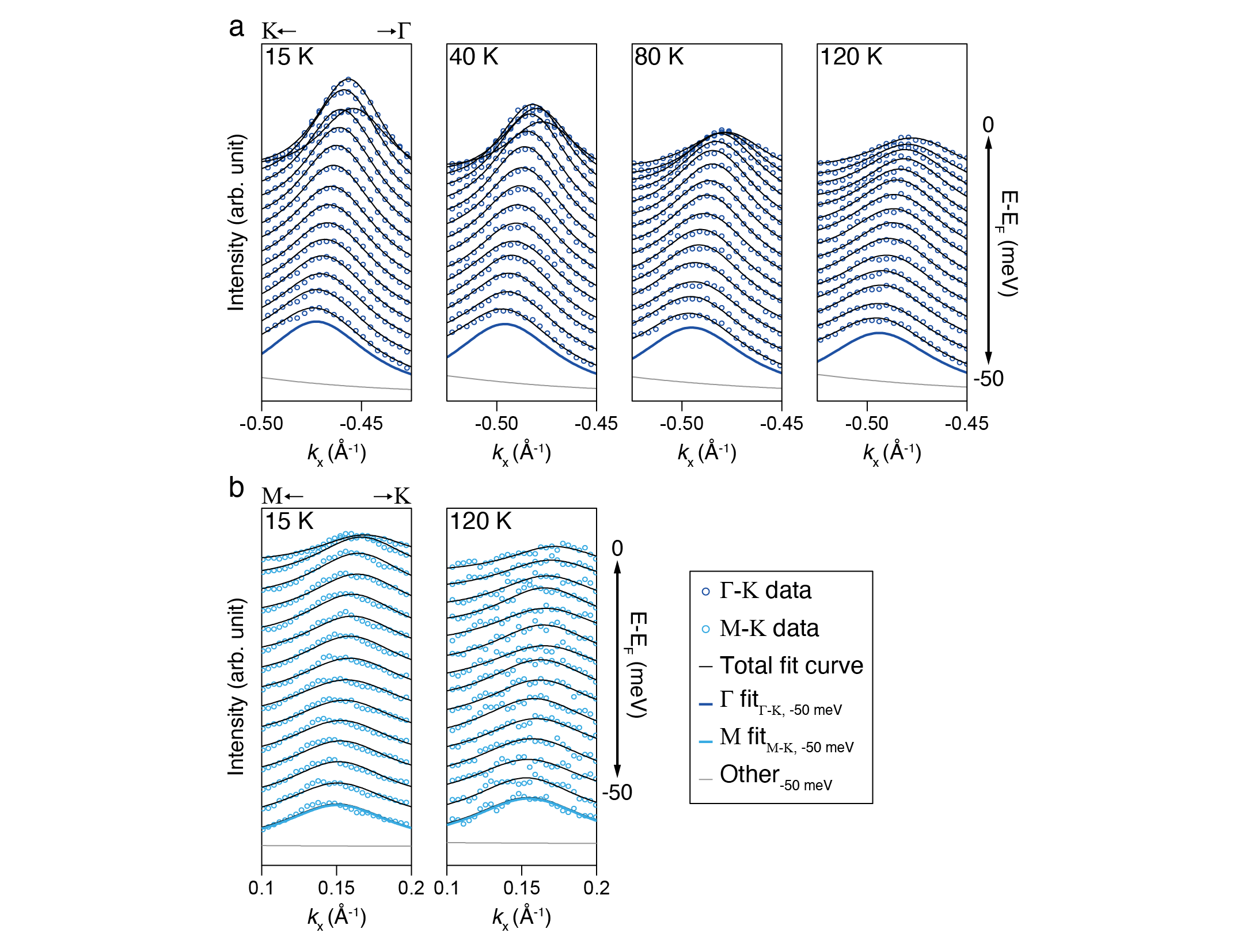


Supplementary Figure S4.

a, b) MDC fitting results of Pd_0.12_TaSe_2_ at the Γ-K (a) and M-K (b) high symmetry lines with various temperatures and binding energies. Dark/light blue empty circles are the raw data, black solid lines are total fit curve including all necessary terms. Dark/light blue solid lines are single-peak fittings for each spectrum, and gray lines are additional peaks where the peak positions locate outside of the plotted momentum range. The energy step of MDC stack is approximately 3.6 meV; some data points in both energy and momentum directions are not plotted for the better visualization.


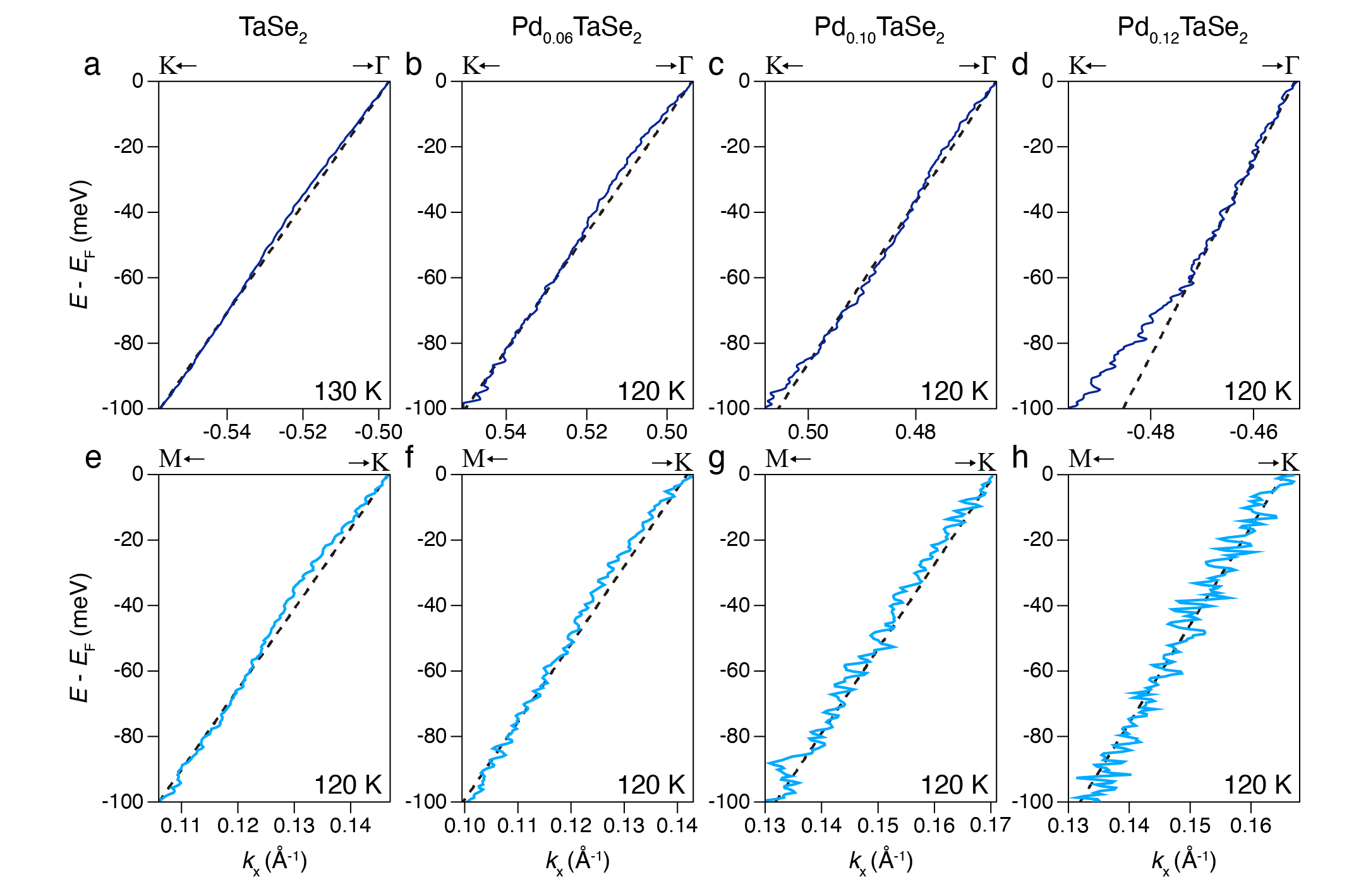


Supplementary Figure S5.

a–h) Peak positions for each intercalation level at the highest temperature obtained by MDC fitting (colored solid lines) and the estimated bare band (dashed lines) along the Γ-K (a to d) and M-K (e to h) high symmetry lines.


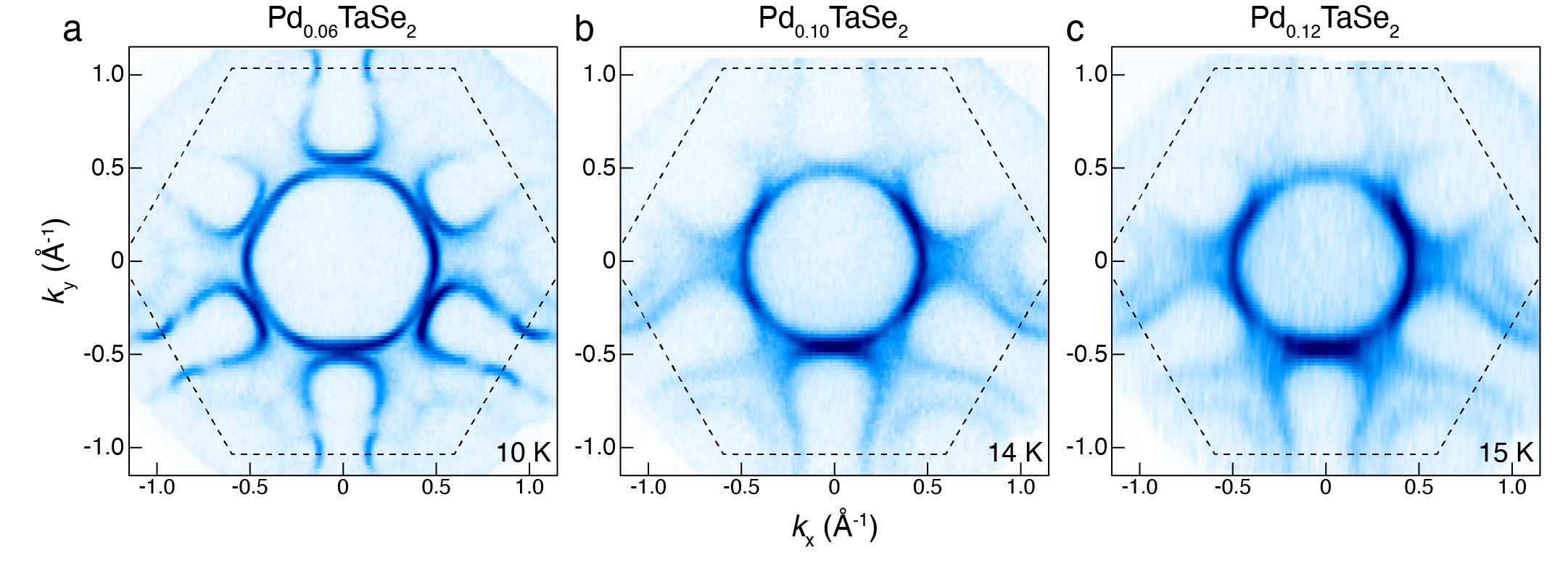


Supplementary Figure S6.

a–c) Fermi surfaces of 2*H*-Pd_0.06_TaSe_2_ (a), 2*H*-Pd_0.10_TaSe_2_ (b) and 2*H*-Pd_0.12_TaSe_2_ (c).


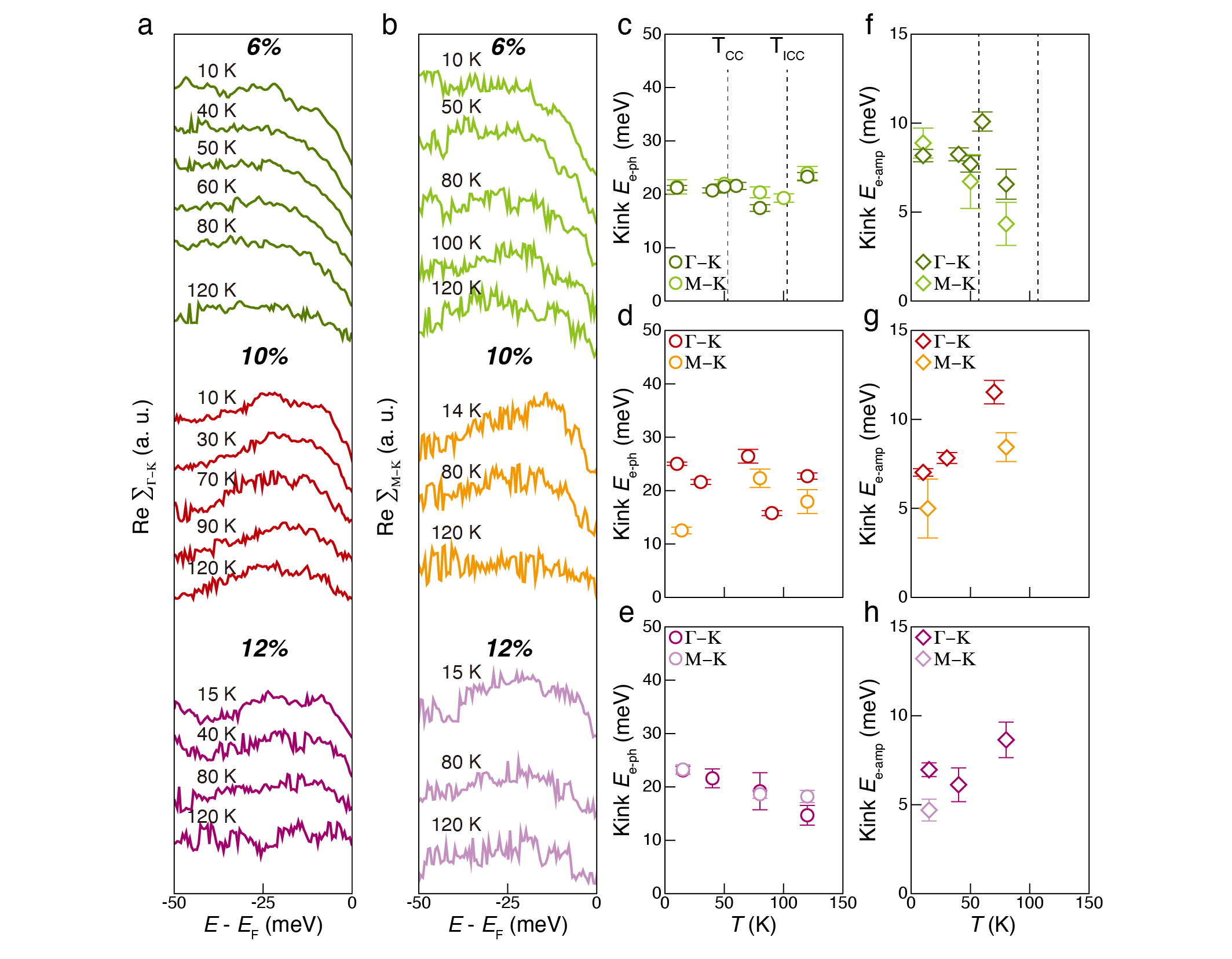


Supplementary Figure S7.

a, b) Temperature- and intercalation-dependent real part of the self-energies for Γ-K (a) and M-K (b), plotted with offsets. c–h) Temperature dependence of the e-ph (c-e) and e-amp (f-h) kink energies obtained from fitting to a continuous function consists of several linear lines (refer Figure S11).


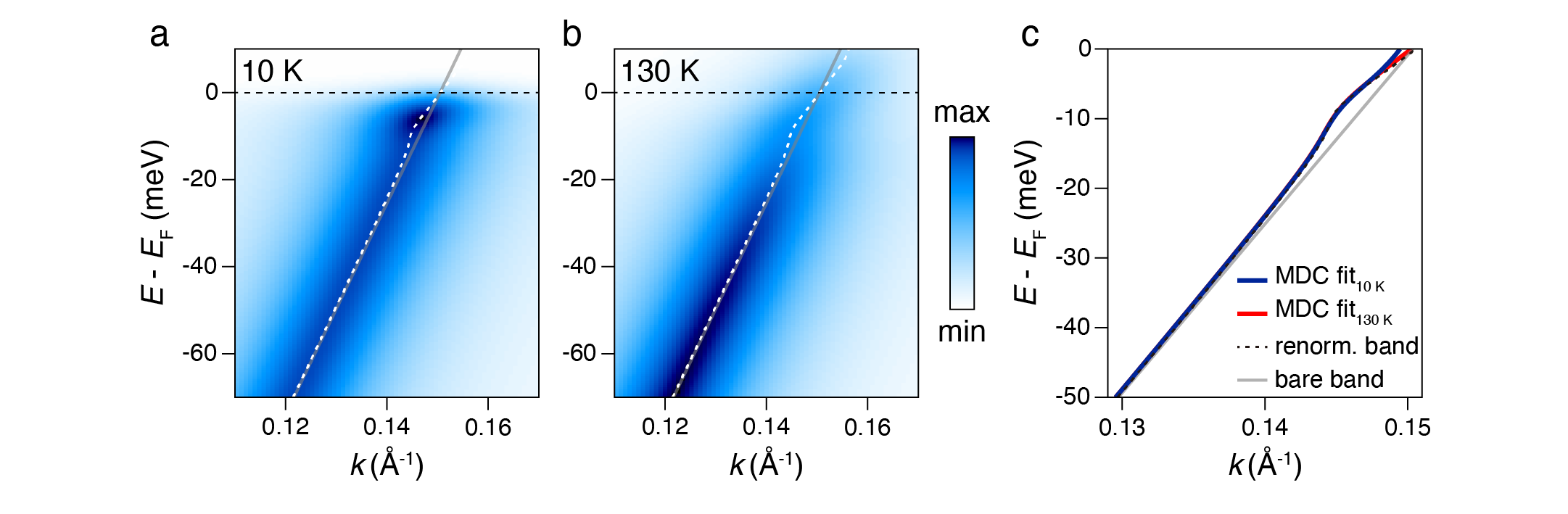


Supplementary Figure S8.

a, b) Simulated spectra for 10 K (a) and 130 K (b). Other than the thermal broadening effect, all the parameters are kept. The dashed lines are peak positions with kink (renormalized band dispersions), while the gray solid lines are bare band dispersions. c) Peak positions of (a) and (b) obtained by the MDC fitting of the simulated spectra.

Supplementary Figure S9.

a) Simulated band dispersions with 24.5 meV kink at various temperatures. A Debye spectrum is used for the Eliashberg coupling function. b) Temperature dependent coupling constant of the simulated dispersions with different *A* values, the weight constant in the Debye spectrum.


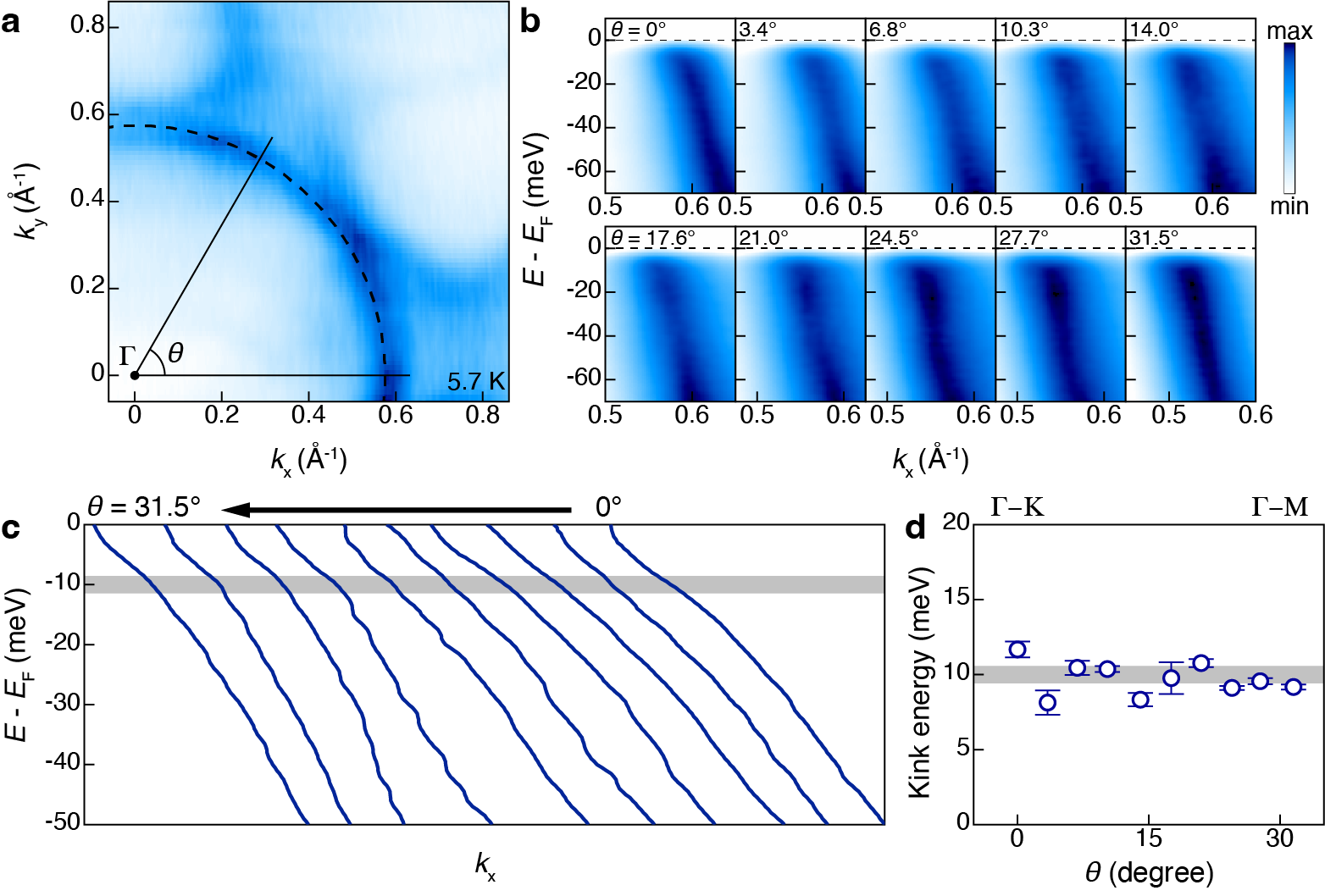


Supplementary Figure S10.

a) Fermi surface of 2*H*-TaSe_2_ obtained by ARPES. Dashed line is a Γ-band guide for the eye. b-d) Momentum-dependent ARPES spectra (b), band dispersion extracted from the MDC fitting (c), and the lowest kink energy (d) of the Γ band.


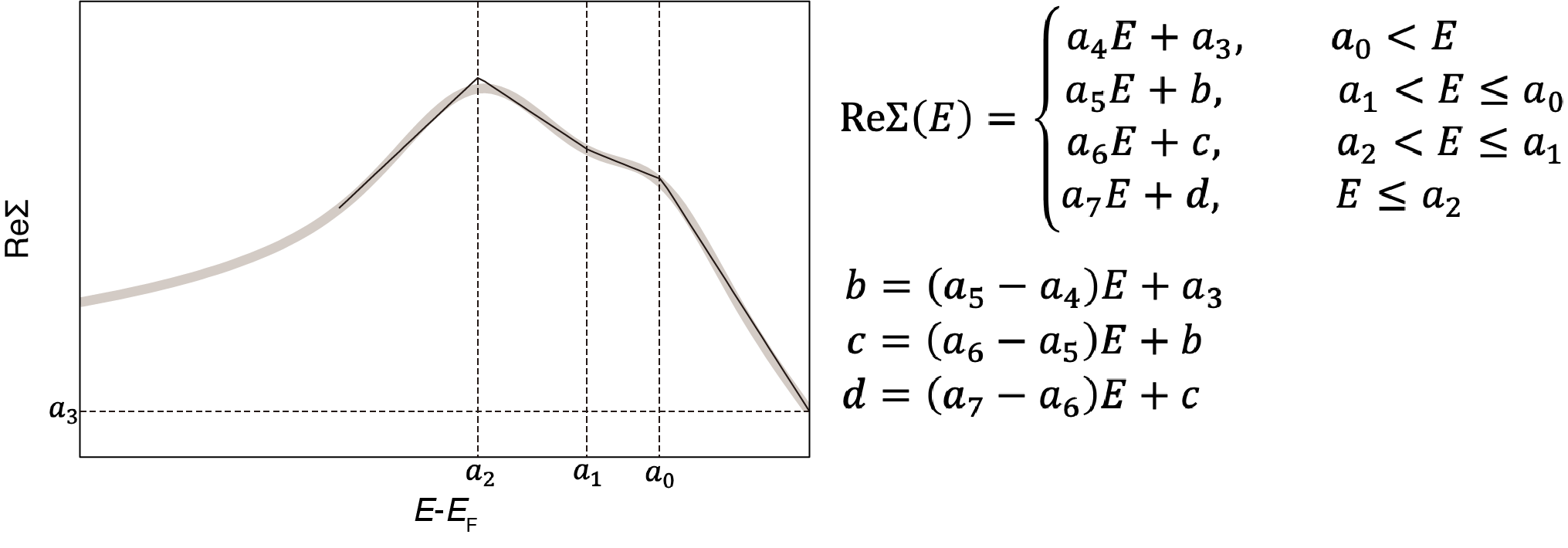


Supplementary Fig. S11.

The way to separate two kinks. The real part of the self-energy is fitted with a continuous function consisting of multiple linear lines. The vertices $a_{0}$ and $a_{2}$ are defined as kink energies, and the slopes $a_{4}$ and $a_{6}$ are used to calculate coupling constants.


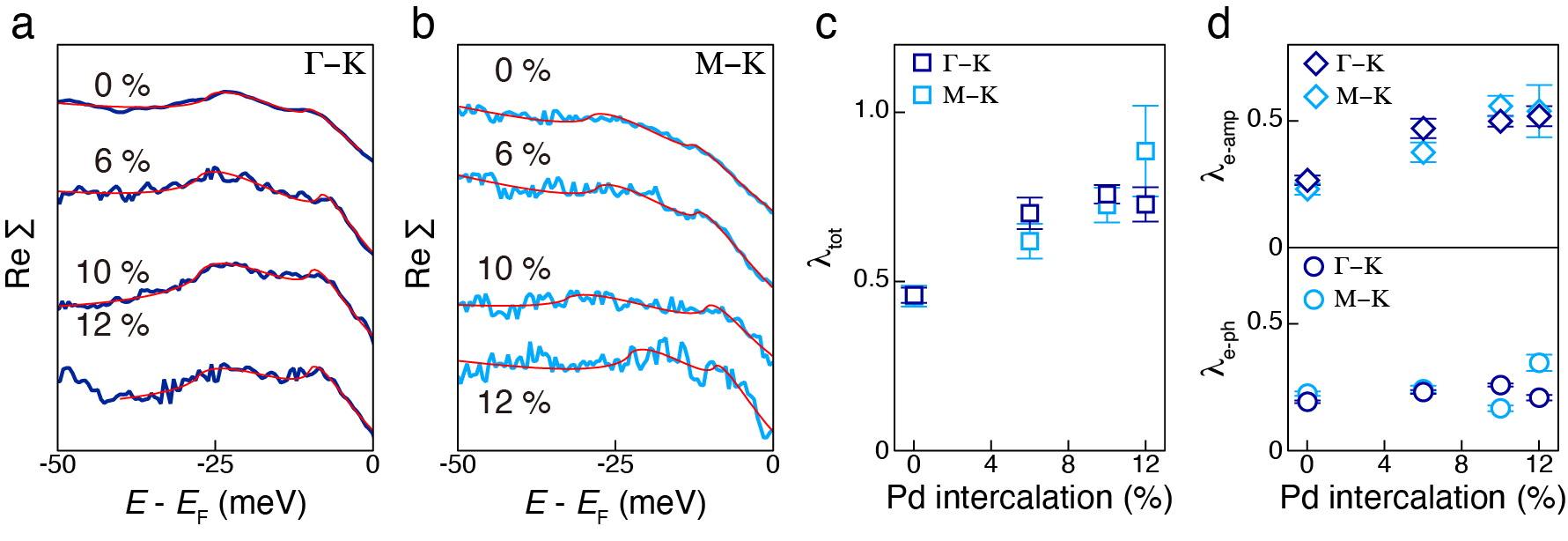


Supplementary Figure S12.

a, b) Intercalation dependence of the real part of the self-energies, fitted with the Debye model (red curves). c, d) Intercalation dependence of the $\lambda_{tot}$ (squares), $\lambda_{e-amp}$ (diamonds), and $\lambda_{e-ph}$ (circles).


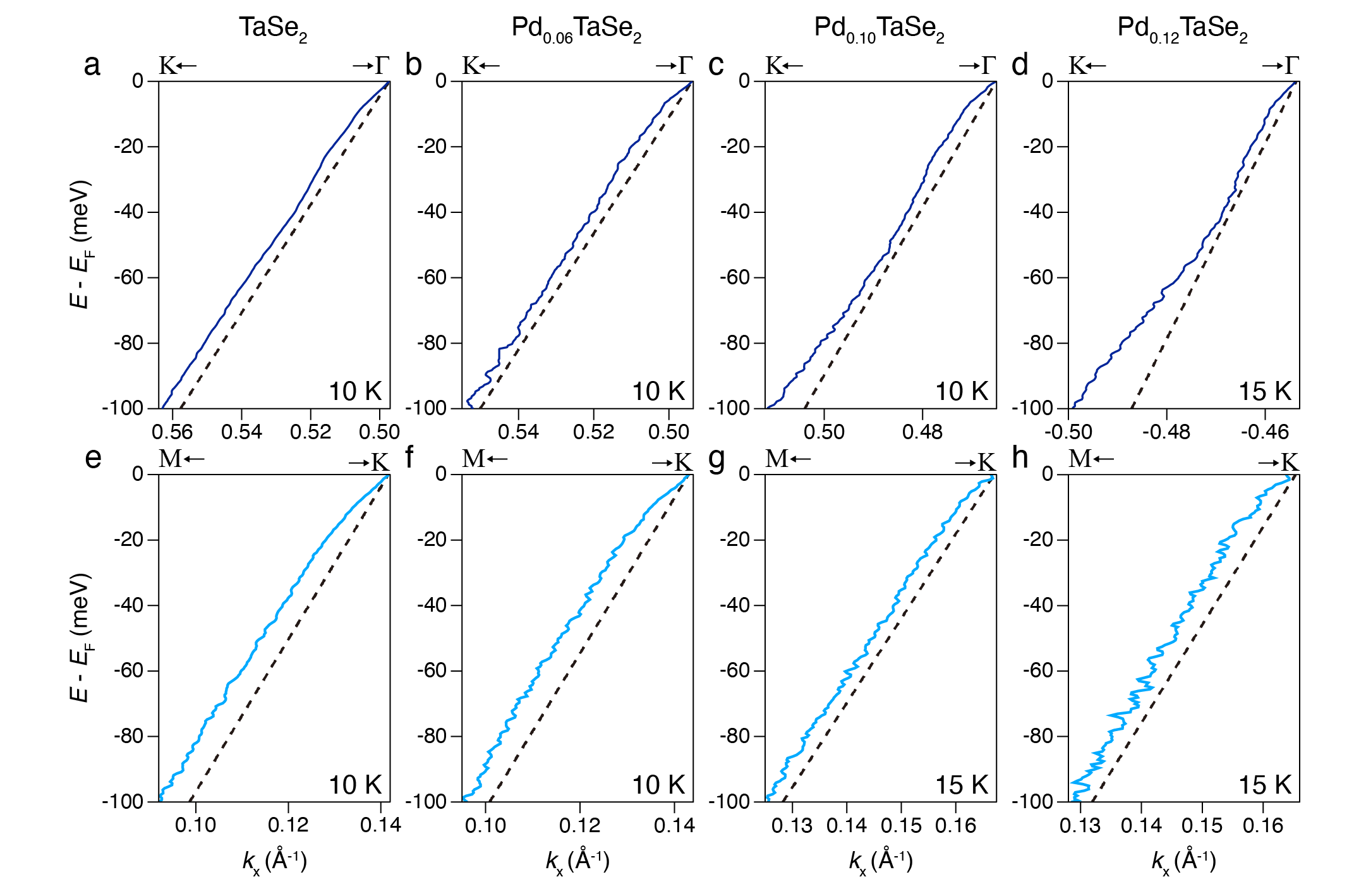


Supplementary Figure S13.

a-h) Peak positions for each intercalation level at the lowest temperature obtained by the MDC fitting (colored solid lines) and the estimated bare band (dashed lines) at the Γ-K (a-d) and the M-K (e-h) high symmetry lines.


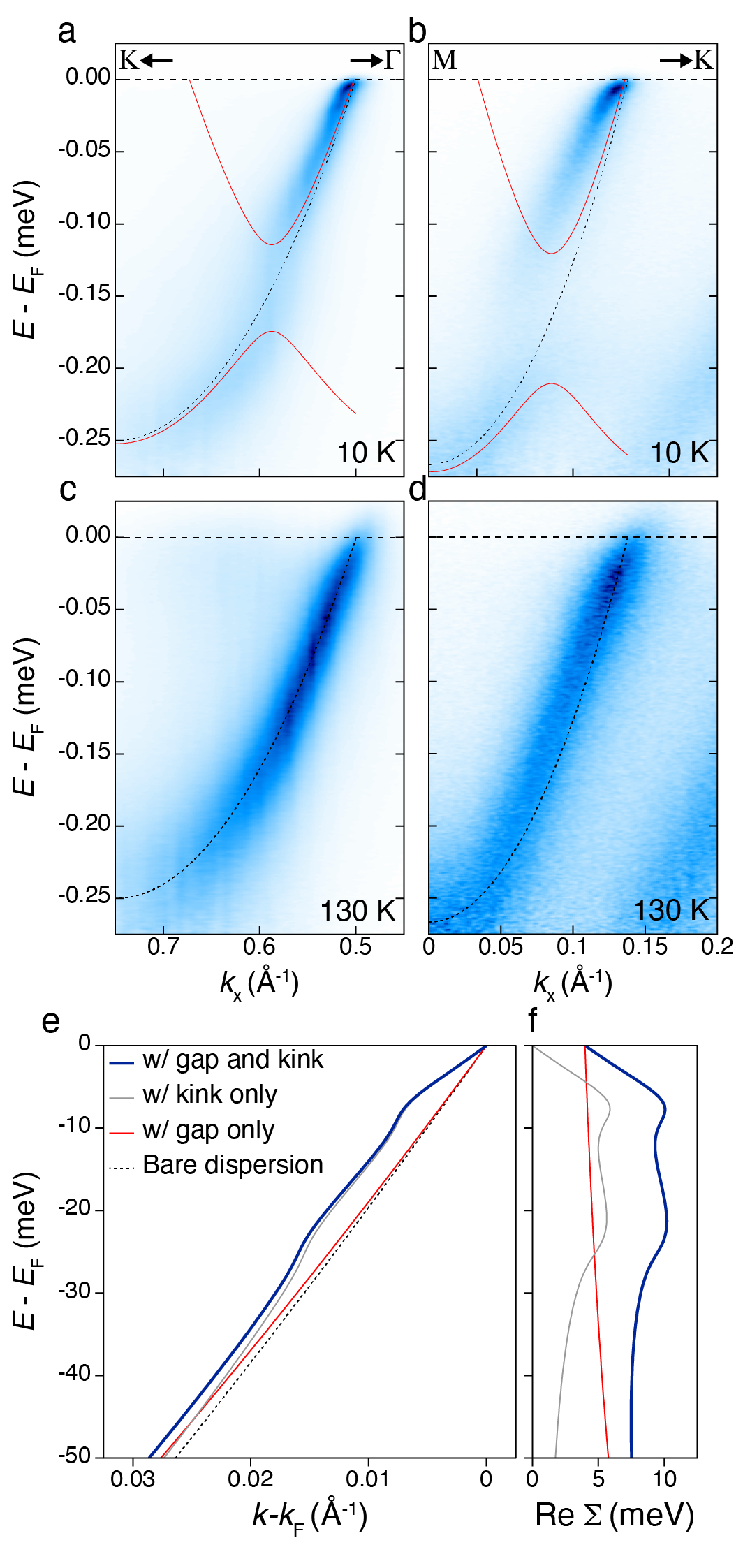


Supplementary Figure S14.

a-d) The ARPES spectra of 2*H*-TaSe_2_ at the CCDW phase (a, b) and the normal phase (c, d) with band calculation. Dashed lines in (a) to (d) are the parabolic bare band dispersions, and red solid lines in (a) and (b) are the gapped dispersions. e, f) Magnified image of the calculated band dispersions (e) and the corresponding real part of the self-energy (f), with and without kinks and a hybridization gap.


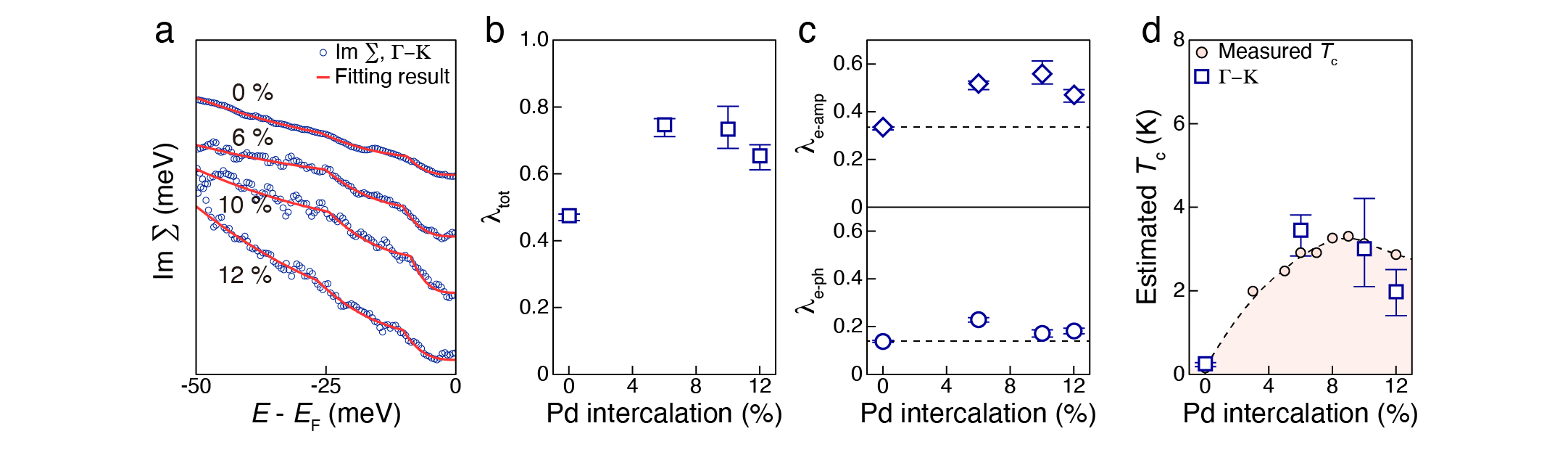


Supplementary Figure S15.

a) Intercalation dependence of the imaginary part of the self-energy (blue empty circles) and its fitting results (red solid lines). b, c) Intercalation dependence of total, e-amp, and e-ph coupling constant extracted from the fitting. d) Estimated *T*_c_ with the McMillan’s equation. Coulomb pseudopotential μ* is assumed to be constant value 0.18. and the Debye temperature Θ_D_ is inserted with the average of the e-ph and e-amp kink energies. The filled circles are the *T*_c_ obtained by the transport measurement in ref. [13] and the dashed line is a guide.


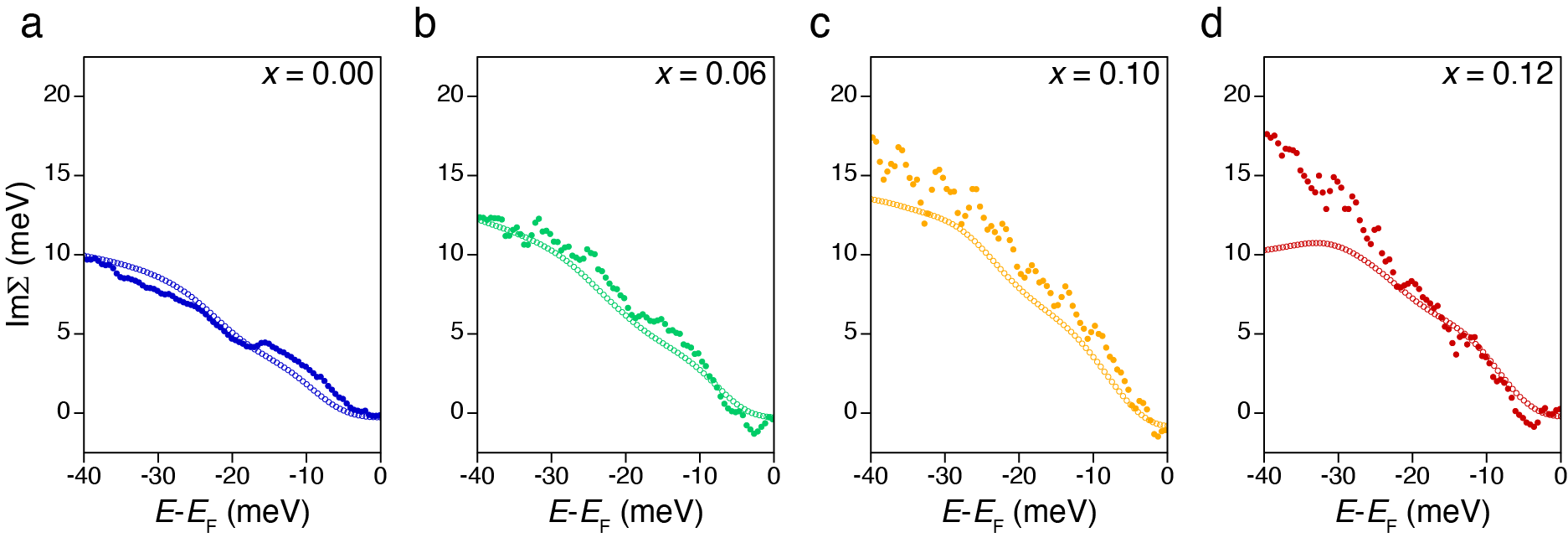


Supplementary Figure S16.

a-d) The imaginary part of the self-energies with offsets calculated by two different methods: MDC peak width multiplied by bare Fermi velocity (filled circles), and applying Kramers-Kronig transformation to the real part of the self-energy (empty circles).
